# The Inhibitory Effect of Sulforaphane on Bladder Cancer Cell Depends on GSH Depletion-Induced by Nrf2 Translocation

**DOI:** 10.3390/molecules26164919

**Published:** 2021-08-13

**Authors:** Canxia He, Luigina P. Buongiorno, Wei Wang, Jonathan C. Y. Tang, Natalizia Miceli, Maria Fernanda Taviano, Yujuan Shan, Yongping Bao

**Affiliations:** 1 Department of Preventative Medicine, Zhejiang Provincial Key Laboratory of Pathological and Physiological Technology, Medical School of Ningbo University, Ningbo 315211, China; hecanxia@nbu.edu.cn; 2 Norwich Medical School, University of East Anglia, Norwich NR4 7UQ, UK; luiginap.buongiorno@gmail.com (L.P.B.); wei.wang@uea.ac.uk (W.W.); Jonathon.Tang@uea.ac.uk (J.C.Y.T.); 3 Department of Chemical, Biological, Pharmaceutical and Environmental Sciences, University of Messina, Viale Palatucci, 98168 Messina, Italy; nmiceli@unime.it (N.M.); mtaviano@unime.it (M.F.T.); 4 School of Public Health and Management, Wenzhou Medical University, Central Northern Road, Wenzhou 325035, China

**Keywords:** sulforaphane, bladder cancer, glutathione, Nrf2, chemoprevention

## Abstract

Sulforaphane (SFN), an isothiocyanate (ITCs) derived from glucosinolate that is found in cruciferous vegetables, has been reported to exert a promising anticancer effect in a substantial amount of scientific research. However, epidemical studies showed inconsistencies between cruciferous vegetable intake and bladder cancer risk. In this study, human bladder cancer T24 cells were used as in vitro model for revealing the inhibitory effect and its potential mechanism of SFN on cell growth. Here, a low dose of SFN (2.5 µM) was shown to promote cell proliferation (5.18–11.84%) and migration in T24 cells, whilst high doses of SFN (>10 µM) inhibited cell growth significantly. The induction effect of SFN on nuclear factor (erythroid-derived 2)-like 2 (Nrf2) expression at both low (2.5 µM) and high dose (10 µM) was characterized by a bell-shaped curve. Nrf2 and glutathione (GSH) might be the underlying mechanism in the effect of SFN on T24 cell growth since Nrf2 siRNA and GSH-depleting agent L-Buthionine-sulfoximine abolished the effect of SFN on cell proliferation. In summary, the inhibitory effect of SFN on bladder cancer cell growth and migration is highly dependent on Nrf2-mediated GSH depletion and following production. These findings suggested that a higher dose of SFN is required for the prevention and treatment of bladder cancer.

## 1. Introduction

Bladder cancer (BC) is the ninth most common cancer worldwide, with an estimated 550,000 new cases and 200,000 deaths in 2018, and the incidence of this disease increases with age [1]. Approximately 75% of newly diagnosed BCs are noninvasive, and more than half of them have recurrence and progression despite local surgery; the remaining 25% of the patients present with muscle invasion and often have poor outcomes despite systemic therapy [2].

Isothiocyanates (ITCs) are a class of well-known cancer-prevention phytochemicals derived from glucosinolates found in cruciferous vegetables such as broccoli, cauliflower and Brussel sprouts. The most extensively studied ITCs, for its protective effects demonstrated in various cell culture systems and animal models, is SFN (4-methylsulfinylbutyl isothiocyanate). Results from epidemiologic studies on BC incidence and survival support a protective effect of cruciferous vegetable intake. For example, a strong and significant inverse association was observed between BC mortality and broccoli intake in a total of 239 BC patients after an average of 8 years of follow-up [3]. In a prospective study involving 47,909 men over 10 years, cruciferous vegetable consumption was inversely associated with BC risk [4]. In another hospital-based case-control study involving 275 patients with primary BC and 825 individuals without cancer, there was a strong and statistically significant inverse association between the risk of BC and intake of raw cruciferous vegetables [5]. However, the results from epidemiologic studies were inconsistent. A meta-analysis of five cohorts and five case-control studies suggested that only a high intake of cruciferous vegetables was associated with the reduced risk of bladder cancer [6]. A prospective population-based cohort study of 82,002 Swedish women and men showed that no associations were observed between cruciferous vegetables and bladder cancer incidence [7]. In another prospective cohort study, there was also no association observed between cruciferous vegetable intake and bladder cancer risk in 27,111 male smokers aged 50–69 years over a median of 11 years follow-up [8]. Results from a meta-analysis of prospective cohort studies of 14 cohorts with 17 studies including 9447 cases also suggested that there was no correlation between cruciferous vegetable intake and bladder cancer risk [9].

Despite mixed outcomes from epidemiologic studies, reports from animal studies have confirmed that SFN or SFN-containing broccoli sprout extract inhibit carcinogenesis, cancer development and/or progression in a wide variety of organs, including breast, colon, liver, stomach, prostate, and especially bladder [10,11,12,13,14,15,16]. Our previous results, both in vitro and in vivo, have also confirmed the inhibitory effect of SFN on bladder cancer [14,17,18,19].

The inhibitory effect of SFN on BC depends on its metabolic characteristic. After oral ingestion, ITCs are rapidly absorbed, metabolized, and almost exclusively excreted and concentrated in the urine. The most abundant ITCs metabolite is selectively accumulated in bladder tissue as NAC-ITC metabolite (mercapturic acid). As a result, the bladder epithelium, where the majority of the bladder cancer originates (90–95%), is the most exposed tissue to the ITCs and their metabolites in vivo, due to the physiological storage of urine containing NAC-ITCs by bladder [20]. The metabolic characteristic suggests that dietary SFN is highly effective in defending against cancer in the bladder than in any other target organs [21]. The chemopreventive effects of the ITCs are traditionally attributed to their ability to prevent tumorigenesis through enhancement of carcinogen detoxification by phase 2 detoxification; induction and the blocking of carcinogen activation by phase 1 inhibition; ITCs also inhibit tumor formation regulating cell proliferation and controlling cell migration [22]. The induction of phase 2 enzymes is strictly related to the translocation of the nuclear factor NF-E2–related factor 2 (Nrf2) into the nucleus. Epidemiological studies aim to reveal the association between vegetable intake and disease risk in a population. These findings from epidemiological studies indicate a promising anticancer effect of SFN on BC, although there are some inconsistencies.

Here, in this study, human bladder cancer T24 cell was used as an in vitro model in this study for maximizing the beneficial effects and revealing the potential of mechanism of SFN on cancer prevention or treatment therapy. Our results suggested that the inhibitory effect of SFN on cell proliferation and migration is highly dependent on the level of GSH depletion and the following production by Nrf2 translocation.

## 2. Results

### 2.1. Effect of SFN on Cell Growth and Migration in T24 Cells

**Figure 1:**
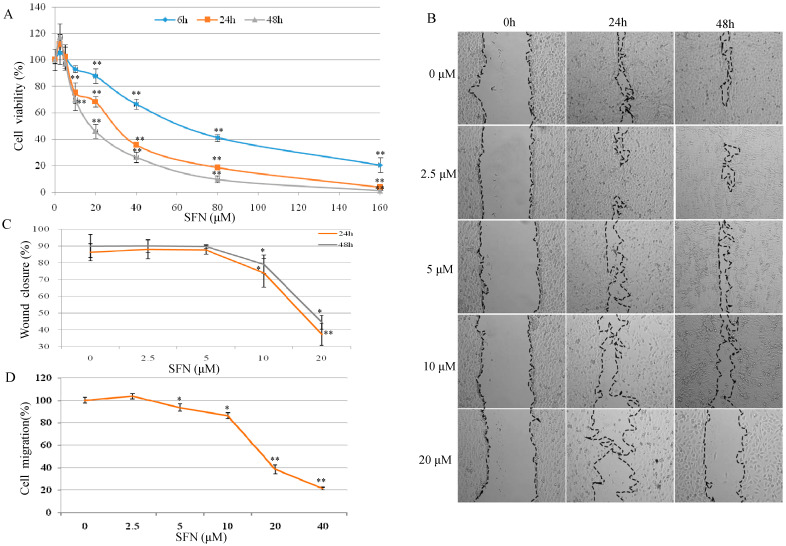
Effects of SFN on cell viability and cell migration in T24 cells. (**A**) T24 cell viability was determined by MTT cell proliferation assay. T24 cells were treated with serial concentrations of SFN (2.5–160 µM) for 6, 24, and 48 h. Each data point represents the mean ± standard deviation (SD) of three experiments, and each treatment was performed in six replicates. (**B**) Scratch assay. A plastic tip was used to scratch a clean wide wound area. Cells were then incubated with SFN for 24 and 48 h. Migration areas were photographed (×100) and calculated with Image J software (**C**). (**D**) Effects of SFN on cell migration. After starvation overnight, T24 cells were treated with SFN (0–40 μM) for 24 h. Cell migration was measured by cell migration assay. Results were compared to control. All data represent the mean ± SD of three experiments, each treatment in six replicates. Statistical significance versus control: * *p* < 0.05, ** *p* < 0.01.

As shown in Figure 1A, T24 cell proliferation was increased by 5.18–11.84% after 6–48 h SFN (2.5 µM) treatment. SFN was shown to have a significant inhibitory effect on T24 cell growth, particularly at concentrations between 10–160 µM after 24 and 48 h treatment.

In Figure 1B, SFN at 2.5 µM slightly increased cell migration in contrast with the control group. After treatment with SFN (10 and 20 µM) for 24 and 48 h, cellular migration was inhibited significantly (Figure 1C). As shown in Figure 1D, SFN at 2.5 µM increased cell migration but not in a significant percentage in comparison with the control cells. SFN from 5 to 40 µM reduced the cell migration in a dose-dependent manner, with the most significant reduction observed at 20 and 40 µM, where cell migration was reduced by 60 and 80%, respectively.

### 2.2. Effect of SFN on Nrf2 Expression and Cell Growth in T24 Cells

**Figure 2:**
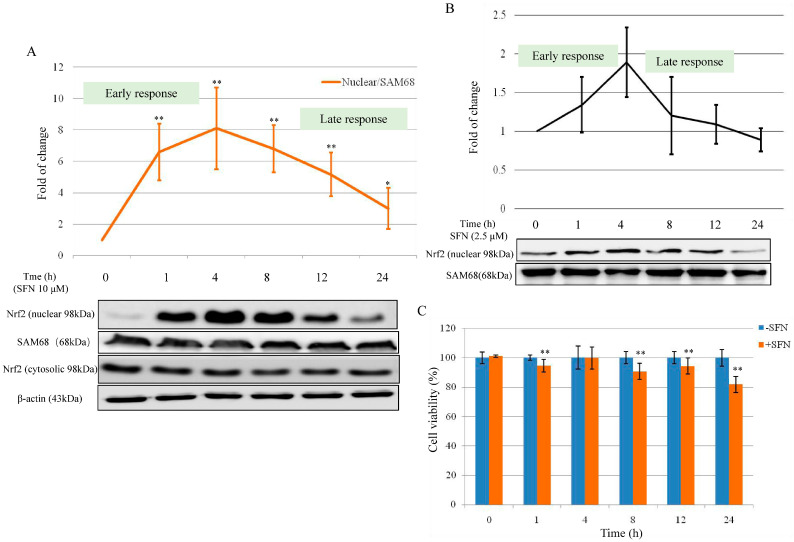
Effect of SFN on Nrf2 expression and cell viability. (**A**) Effect of SFN (10 µM) on Nrf2 nuclear and cytosolic expression after treatment from 0 to 24 h. SAM68 was used as loading control for the nuclear fraction, β-Actin was used as loading control for the cytosolic fraction. (**B**) Nrf2 nuclear expression after SFN 2.5 µM treatment from 0 to 24 h. SAM68 was used as a loading control for the nuclear fraction. (**C**) T24 cell viability was tested after treatment with 10 µM SFN from 0 to 24 h. All data represent the mean ± SD of three experiments in which each treatment was performed in six replicates. Statistical significance versus control: * *p* < 0.05, ** *p* < 0.01.

Since the multi-functions of SFN primarily through Nrf2, we analyzed Nrf2 expression after treatment with serial concentrations of SFN. Compared with Nrf2 cytosolic protein expression (Figure 2A), Nrf2 nuclear expression increased significantly after SFN 10 µM treatment (for 1–4 h). The maximum value of Nrf2 nuclear protein was observed after 4 h treatment (6.52-fold vs. control), then went down to 2.40-fold after 24 h treatment. Results in Figure 2A showed a particularly high induction of Nrf2 nuclear protein after SFN 10 µM in the early response (1 and 4 h). Nrf2 nuclear fraction resulted in a significant increase of 6.60 ± 1.80-fold compared to the control after 1 h treatment with SFN (10 µM), and the maximum value of 8.10 ± 2.60-fold was observed after 4 h treatment. Treatment of 8–24 h SFN (10 µM) also showed induction of nuclear Nrf2, but at a slower rate. The values at 24 h were 3.60 ± 1.30-fold higher than the control (time 0). The analysis of both the cytosolic and the nuclear fractions of Nrf2 showed (Figure 2A) that the induction of nuclear Nrf2 did not result in a corresponding increase in cytosolic Nrf2 expression. This observation confirms the active translocation of Nrf2 from the cytosol into the nucleus.

In Figure 2B, Nrf2 nuclear protein expression was slightly increased after 2.5 µM SFN treatment in early response (1, 4, and 8 h). The maximum value of Nrf2 nuclear protein was observed after 4 h treatment (1.89 ± 0.45 vs. control). After 8 h treatment, the induction effect of SFN (2.5 µM) on Nrf2 nuclear protein expression went down to 1.09 ± 0.25 for 12 h and 0.90 ± 0.13 for 24 h. Between 0 and 4 h (early response), SFN (both 2.5 and 10 µM) increased the nuclear Nrf2 expression in a time-dependent manner; the late response was characterized by the induction of Nrf2 as well.

To determine whether the early and late response of SFN on Nrf2 activation in relation to cell growth, cell viability was tested. In Figure 2C, cell proliferation was decreased by 5.45 to 18.11% when compared with the counterparts after treatment with SFN (10 µM) for 0–24 h.

### 2.3. Effect of SFN on the Cellular Glutathione (GSH) Level in T24 Cells

**Figure 3:**
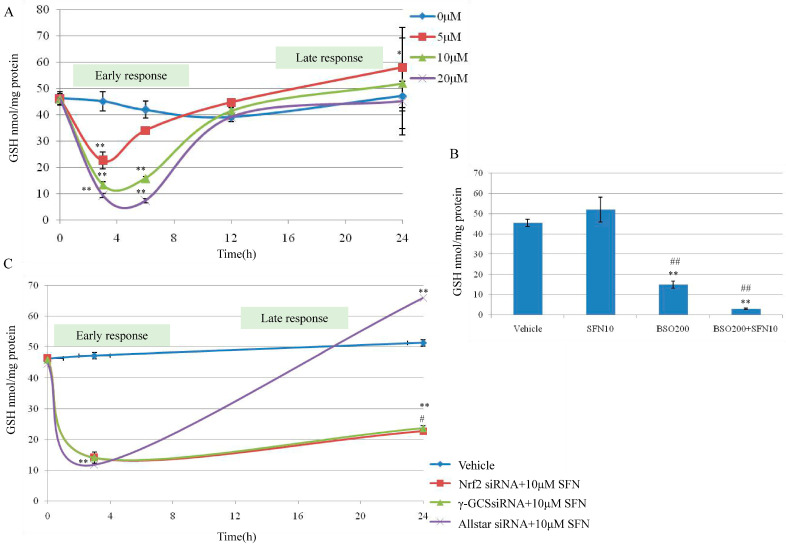
Effect of SFN on GSH synthesis by targeting Nrf2 and γ-GCS in T24 cells. (**A**) Cellular GSH concentrations in T24 cells exposed to SFN. Subconfluent T24 cells were treated with SFN 5–20 µM. At the indicated time (0–24 h), control cells and those treated with SFN 5–20 µM were derivatized and the samples analyzed by HPLC. The significant difference was reported * *p* < 0.05, ** *p* < 0.01. (**B**) Cellular GSH levels in T24 cells exposed to L-Buthionine-sulfoximine (BSO) and SFN. T24 cells were incubated with dimethylsulphoxide (DMSO, vehicle) as control, SFN 10 µM and BSO 200 µM in cotreatment and separately, for 24 h. GSH level was evaluated using HPLC method and has been expressed as nmol/mg of proteins. Significantly difference in GSH concentrations monitored in untreated cells (vehicle), ** *p* < 0.01; significantly difference in GSH concentrations monitored in SFN treated cells, ## *p* < 0.01. (**C**) Effect of SFN (10 µM) treatment on GSH level in Nrf2 and γ-Glutamylcysteine Synthetase (γ-GCS) suppressed T24 cells. T24 cells were treated with Nrf2 siRNA or γ-GCS siRNA, then treated with SFN (10 µM) for 3 h and 24 h. No transfected cells with DMSO (final concentration 0.1%) for 3 h and 24 h were used as a vehicle. Cells transfected with AllStar siRNA and then treated with SFN 10 µM for 3 h and 24 h were used as negative control. Results are mean ± SD of 3 samples. A significant change from basal level is indicated with ** *p* < 0.01, # *p* < 0.05 is significantly different in GSH level at 24 h for Nrf2 siRNA and γ-GCS siRNA treatment compared with the AllStar group.

Cellular reduced GSH affects the accumulation of SFN in cells. It led us to examine whether the potency of SFN was similarly affected. The cells were exposed to increasing concentrations of SFN (5–20 µM) and exposure times (0–24 h). T24 cells were treated with SFN 5–20 µM. As shown in Figure 3A, the concentration of total GSH in T24 cells at time 0 was 58.11 ± 0.55 nmol/mg. When T24 cells were treated with SFN for up to 24 h, the GSH concentration decreased in a time-dependent manner and reached a nadir of 22.33 ± 3.30; 13.06 ± 1.50 and 9.54 ± 0.81 nmol/mg between 3–6 h respectively after 5, 10 and 20 µM of SFN treatments (Figure 3A) and gradually increased to the control level at 12 h and 5–10 nmol/mg higher than the control at 24 h. Our results have demonstrated a completely different effect of SFN; at all the doses tested (5–20 µM), between the short exposition time (0–12 h, early response) and the long exposition time (12–24 h, late response). From this result, the transient down-regulation of GSH, observed between 0–6 h (early response), can be associated with the formation of SFN-GSH adducts. After the transient decrease of intracellular GSH (0–6 h), the GSH level was reestablished within 12 h, and above at 24 h. The concomitance between the down-regulation of GSH level (0–6 h) and the strong induction of Nrf2 (0–4 h) suggests a probable connection between the two events.

### 2.4. Effect of γ-GCS on SFN-Induced GSH Increase

Given that SFN is capable of stimulating GSH synthesis in T24 cells, we tested whether the mechanism involved was dependent on γ-GCS induction; a key enzyme in glutathione biosynthesis regulated at the transcriptional level by Nrf2. To this aim, cells were co-treated with the SFN (10 µM) and a GSH-depleting agent BSO, a specific inhibitor of γ-GCS at 200 µM. The results showed that BSO alone was able to reduce significantly intracellular GSH content by 75% while SFN treatment alone increased GSH content. Interestingly, BSO and SFN co-treatment reduced GSH level by 95% at 24 h. These results support the main role of γ-GCS in SFN-induced GSH increase at 24 h (Figure 3B).

As is known, Nrf2 can regulate the expression of more than 200 genes that contain an antioxidant response element (ARE) in their promoter region, such as phase 2 enzymes, redox-active proteins, GSH-related enzymes, and several other novel enzymes recently identified [23]. Next, since we found that SFN induced the activation of Nrf2, we decided to knock out Nrf2 and evaluated the effect on GSH. To further understand the molecular mechanism through which ITCs modulate GSH level, we knocked down the expression of Nrf2 and γ-GCS in T24 cells. Nrf2 and γ-GCS silencing in T24 cells resulted in a severe depletion of GSH concentration after 3 h treatment with SFN 10 µM (early response); this depletion, as shown in Figure 3C, was significant (*p* < 0.01) compared with the control but not significant if compared with the negative control (AllStar transfected cells). A significant difference in GSH concentration was evident after SFN 10 µM treatment for 24 h (late response), in fact, at this time point (24 h), the GSH concentrations, after siNrf2 or siGCS transfection and treatment with SFN 10 µM, appeared significantly lower compared to control cells transfected with AllStar and treated with SFN 10 µM as well (Figure 3C). These results showed the effective capacity of SFN (10 µM) to reestablish the GSH level within 12 h, and further increased beyond the control after 24 h treatment. The mechanism lies in the activation of Nrf2 translocation and induction of the expression of GSH-related genes, promoting the production of GSH. In contrast, when Nrf2 and γ-GCS were silenced, GSH was not reestablished.

### 2.5. Effect of SFN on UDP-glucuronosyltransferase (UGT) and Cyclooxygenase-2 (COX-2) Expression in T24 Cells

**Figure 4:**
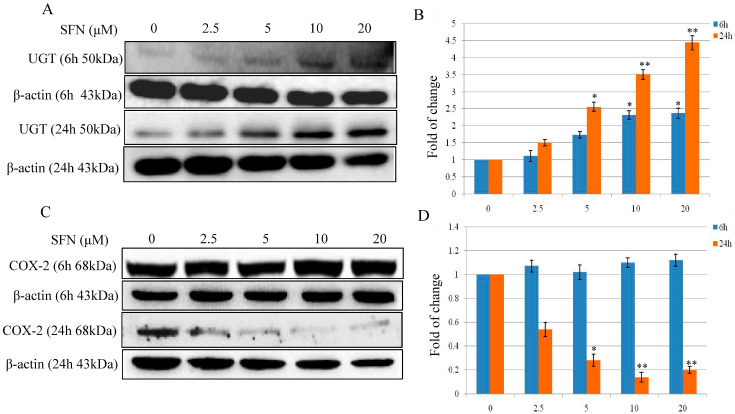
Effect of SFN on UGT and COX-2 expression in T24 cells. (**A**,**B**) Effect of SFN on UGT protein expression after treatment for 6 and 24 h. T24 cells were treated with SFN (2.5, 5, 10 and 20 µM) for 6 and 24 h. (**C**,**D**) Effect of SFN on COX-2 protein expression after treatment for 6 and 24 h. T24 cells were treated with SFN (2.5, 5, 10 and 20 µM) for 6 and 24 h. Data were normalized for β-Actin, and reported as fold variation with respect to the Vehicle group. Statistical significance versus control: * *p* < 0.05, ** *p* < 0.01.

To evaluate the effect of SFN on phase 2 enzyme expression, we measured UGT protein expression after exposing cells to SFN (2.5, 5, 10 and 20 µM) treatment for 6 and 24 h. As shown in Figure 4A, there was a dose-dependent inductive effect of SFN on UGT protein expression. A significant increase was particularly evident when cells were treated with SFN of 5, 10 and 20 µM. When Nrf2 was knocked down using siRNA, the SFN-induced UGT expression was abolished (data not shown).

COX-2, an important enzyme in the synthesis of prostaglandin from arachidonic acid, is inducible in response to cytokines, mitogens, growth factors, and tumor promoters. In the present study, we evaluated the effect of SFN (2.5. 5, 10 and 20 µM) on COX-2 expression in T24 cells after 6 and 24 h treatments. As shown in Figure 4C, COX-2 expression was shown with no significant changes after 6 h treatment (2.5, 5, 10 and 20 µM). As reported in Figure 4C, SFN down regulated COX-2 protein expression in a dose-dependent manner after treatment with SFN (2.5, 5, 10 and 20 µM) for 24 h. A significant decrement of COX-2 expression was evident when cells treated with SFN 10 and 20 µM for 24 h, with a reduction of the protein expression from 49.89 to 90% (Figure 4C,D). However, knockdown of Nrf2 using siRNA did not affect the down-regulation effect of SFN on COX-2 expression (data not shown).

### 2.6. Effect of SFN on Nrf2 Expression and Cell Growth by Targeting γ-GCS

**Figure 5:**
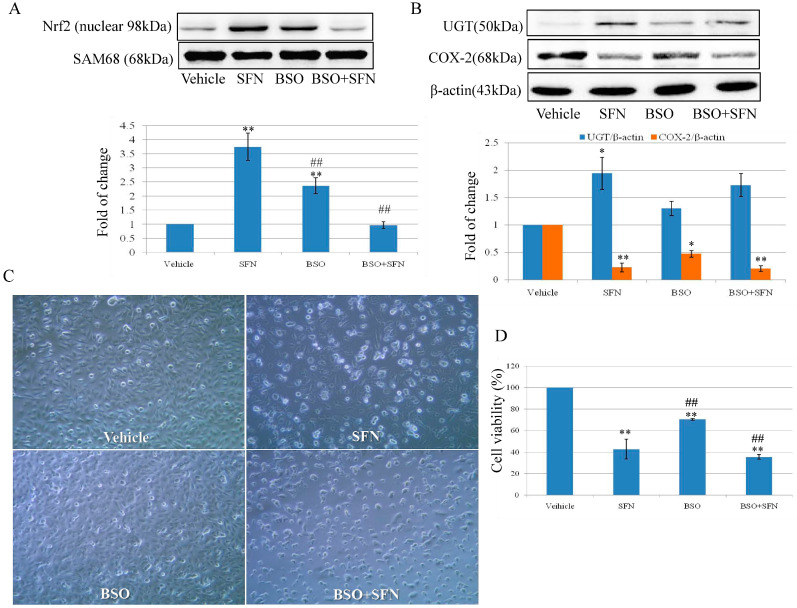
Effect of SFN on the expression of Nrf2 and UGT and cell viability by targeting γ-GCS. T24 cells were incubated with SFN 10 µM and BSO 200 µM in co-treatment and separately for 24 h. Cells were treated with 0.1% DMSO as control. (**A**) Nrf2 nuclear expression in T24 cells exposed to BSO and SFN. SAM68 was used as a loading control. (**B**) UGT and COX-2 expression in T24 cells exposed to BSO and SFN. β-actin was used as loading control. (**C**) After treatment with BSO or SFN, cells were photographed with a microscope (×100). (**D**) After treatment with BSO or SFN, T24 cell viability was determined by MTT cell proliferation assay. ** *p* < 0.01 is represented with a significant difference compared with untreated cells (the Vehicle group). Statistical significance versus SFN treatment: * *p* < 0.05, ## *p* < 0.01.

The results in Figure 3B,C confirmed that the role of γ-GCS in SFN-induced GSH. Here, BSO was used to examine the expression of Nrf2, UGT, and COX-2 and its role in cell proliferation. As shown in Figure 5A, nuclear Nrf2 expression was increased by 3.75-fold after 10 µM SFN treatment, whereas no significant change was observed in the BSO + SFN treatment group when compared with the control group. The expression of UGT was increased by 1.94 and 1.73-fold in SFN and BSO + SFN treatment group. COX-2 expression was decreased by 0.23-fold after 10 µM SFN treatment for 24 h. BSO treatment decreased COX-2 expression by 0.47-fold. In Figure 5D, cell growth was inhibited by 42.84% after SFN treatment, whereas the number was decreased to 35.58% in BSO + SFN treatment group. For BSO treatment only, cell proliferation rate was decreased to 70.47% compared with the control group.

All the results suggest that the effect of SFN on bladder cancer cell growth and migration is probably attributable to Nrf2-mediated GSH production and phase 2 enzyme expression.

## 3. Discussion

The present findings show that low doses of SFN activate cell proliferation and high doses decrease cell viability and migration. Nrf2 activation and GSH level might play a key role in the effect of SFN on cell proliferation. On the basis of these findings, our results imply that a higher dose of SFN is required for the prevention and treatment of bladder cancer.

The epidemiological evidence with respect to the consumption of cruciferous vegetables against bladder cancer is inconsistent. The results from some epidemiological studies suggested that a high ITCs intake is associated with decreased risk of bladder cancer [4,24]. Our previous results from in vitro cell models show that a low dose of SFN promotes several types of cancer cell growth, including liver and colon cancer [23]. A low dose of ITCs that promotes cancer cell growth may help to explain the inconsistent results in epidemiological studies [25]. For the majority of the population, the plasma concentration of ITCs is probably to be much lower than sub-µM. Plasma ITCs can be improved by taking supplements and increasing intake. So, a relatively high dose of SFN is needed to achieve its beneficial effect in cancer chemoprevention or treatment.

Here, the results from cell viability and migration revealed the characteristic bell-shaped curve identified as early and late response effect of SFN on Nrf2 expression (Figure 1). SFN (2.5 and 10 µM) increased nuclear Nrf2 expression in a manner of an early and late response effect (Figure 2A,B). Nrf2 is a major regulator of cell survival [26,27]. Corresponding to the early and late response of SFN on Nrf2 expression, the transient down-regulation of GSH was observed between 0–6 h (early response) and then up-regulation of GSH level during 12–24 h (Figure 3). It was demonstrated that ITCs intracellular accumulation is dependent on the intracellular GSH level [28,29], and also it was demonstrated that SFN rapidly conjugates with GSH causing a transient GSH depletion [30]. However, our results have also shown that after the transient decrease of intracellular GSH (0–6 h), the GSH level was re-established within 12 h, and then increased at 24 h.

Phase 2 enzymes, for example, UGT, is shown characteristically with both an early and late response, which significantly increased after treatment with SFN for 6 and 24 h (Figure 4A). A large body of literature suggests that COX-2 is overexpressed in human bladder cancer and is closely related to the progression, prognosis, and recurrence of bladder cancer [31,32]. COX-2, a key mediator in inflammation, promotes reactive oxygen species (ROS) production and shifts redox state in cells [33]. Here, COX-2 expression shows with late response only after SFN treatment since it is unchanged at the time point of 6 h. Our previous results suggested that p38 MAPK activation, not Nrf2, is essential in SFN-mediated COX-2 expression [19].

**Figure 6:**
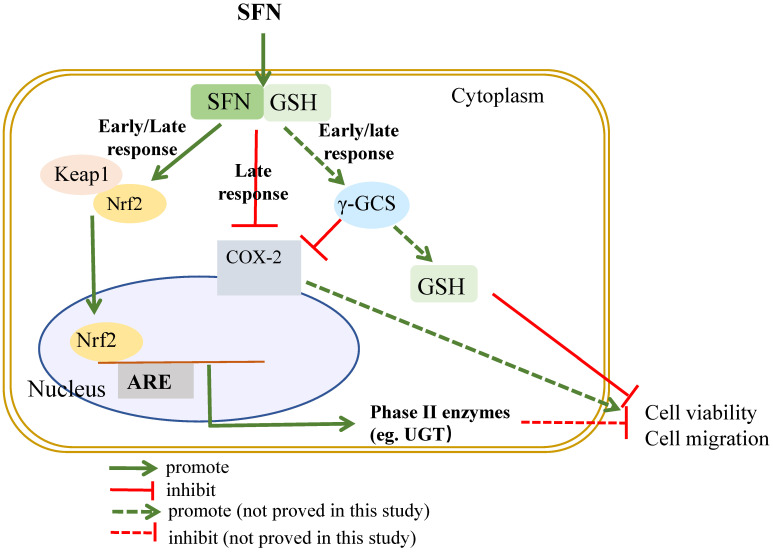
A proposed mechanism of the inhibitory effects of SFN on cell growth and migration.

Results show that Nrf2 expression and its induction effect on GSH is pivotal in the inhibitory effect of SFN on bladder cancer cell growth by using Nrf2 siRNA and GSH-depleting agent BSO (Figure 3 and Figure 5). SFN is able to react with free thiols and consequently, once into the cell, it reacts with glutathione causing a transient intracellular alteration of the GSH:GSSG ratio. This, in turn, produces a redox stress and a marked GSH depletion within the first 4 h after SFN treatment. In terms of eliciting an adaptive response and then Nrf2 activation to such stress, SFN directly forms adducts with cysteines in Keap1 causing a covalent modification of Keap1 that prevents its binding with Nrf2 or indirectly induces oxidative modifications on Keap1 through intracellular GSH depletion [34]. Enzyme γ-GCS, the rate limiting step in GSH synthesis, is regulated at transcriptional level by Nrf2. Nrf2 mediated γ-GCS gene expression leading to elevate GSH levels. Nrf2 once migrates into the nucleus, binds with the ARE sequence and induces the synthesis of the Nrf2 dependent enzymes such as γ-GCS that reestablish the GSH level between 12–24 h, finally, cell growth is inhibited. The mechanistic profile of SFN on cell growth is summarized in Figure 6. The results from Edward and co-workers suggest that Nrf2 related pathway provides an explanation for its dose responses in inflammatory degenerative diseases, which is quite consistent with our findings [35].

In conclusion, our data provide evidence that SFN is a promising and complicated pleiotropic chemopreventive and therapeutic agent for bladder cancer. The chemopreventive effect can be optimally achieved by frequent consumption of isothiocyanates at relatively high concentrations. Nrf2 expression and GSH production may contribute to the inhibitory effect of SFN on bladder cancer cell growth. Further work should be warranted to study the effect of ITCs/SFN on cell growth in vivo for maximizing the beneficial effects and minimizing the potential risks of ITCs in cancer management.

## 4. Materials and Methods

### 4.1. Experiment Reagents

Sulforaphane was purchased from Toronto Research Chemicals (Toronto, Canada). Sodium/Mc selenite, DMSO, BSO, and Bradford reagent were all purchased from Sigma (Dorset, UK). Protease inhibitor cocktail tablets were obtained from Roche Applied Science (UK). RPMI-1640 medium was purchased from Invitrogen Corporation (UK). Antibodies against Nrf2 (Catalog No.13032), Sam68 (Catalog No.333), COX-2 (Catalog No.376861), UGT (Catalog No.271268), β-actin (Catalog No.7210) were all purchased from Santa Cruz Biotechnology (Santa Cruz, Heidelberg, Germany). The nuclear Extraction Kit was purchased from Active Motif^®^ International (UK). Electrophoresis and Western blotting supplies were obtained from Bio-Rad (Hertfordshire, UK). The HiPerfection Transfection Reagent Kit was purchased from QIAGEN^®^ (west Sussex, UK), Nrf-2 siRNA (ID 115764) was obtained from Applied Biosystems (Manchester, UK).

### 4.2. Cell Culture

Human bladder cancer T24 cells were obtained from the European Collection of Cell Cultures (ECACC) and grown in RPMI-1640 medium supplemented with 10% (*v/v*) heat-inactivated fetal bovine serum, 1% Penicillin/Streptomycin (5000 U), and 1% L-Glutamine (200 mM). Cells were grown in a humidified atmosphere (37 °C, 5% CO_2_). In this study, all treatments and controls contained a final DMSO concentration of 0.1%.

### 4.3. Cell Viability Assay

The MTT (3-(4,5-dimethylthiazol-2-yl)-2,5-diphenyltetrazolium bromide) cell proliferation assay was employed to detect the toxicity of SFN (2.5–40 μM) in T24 cells. T24 cells were seeded in a 96 well plate at 5.0 × 10^3^/well, and incubated for 48 h. After being treated and incubated with SFN for 6, 24 and 48 h, MTT reagent 10 µL/100 µL per well (final concentration 0.5 mg/mL, mix with fresh medium) were added to the 96 well plates. Incubate at 37 °C for 1 h. Then, extracted the medium by using a pump and re-suspended with DMSO. The final absorbance in the well was recorded using a microplate reader (BMG Labtech Ltd., Bucks, UK) at a test wavelength of 570 nm and a reference wavelength of 670 nm, and IC_50_ was calculated using software Calcusyn (Biosoft, Cambridge, UK).

### 4.4. Scratch Assay

T24 cells were seeded in 12 well plates at 0.3 × 10^6^ cells/well in a final volume of 1 mL. The day after, when reaching ~90–100% confluence, without changing the medium, gently and slowly, with a 1 mL pipette tip, the monolayer was scratched across the center of the well in a long-axial line creating a gap. To remove the detached cells, each well was gently washed twice with prewarmed medium. The medium containing SFN (2.5–20 μM) or DMSO 0.1% (vehicle), was added. Cells were incubated for 24 or 48 h. Three photographs were made for each well (top, center and bottom). Image J software was used for measuring the scratch width, three measurements for each photograph were made (top, center, and bottom) and results expressed as an average of these three measurements. Results were shown as a percentage of wound closure.

### 4.5. Cell Migration Assay

Cell migration was quantified using a ThinCert cell culture inserts cell migration assay (Greiner Bio-One Ltd., Kremsmünster, Austria). After overnight starvation in serum-free medium, cells were treated with various concentrations of SFN for 24 h, the cells migrating through a PET membrane were labeled fluorescently with Calcein-AM and quantified by microplate reader (BMG Labtech Ltd., Aylesbury, UK) with an excitation wavelength of 485 nm and emission wavelength of 525 nm.

### 4.6. Protein Extraction and Western Blot Analysis

For protein isolation, cells were seeded in a 6-well plate at 1 × 10^5^/mL or 10 cm dishes at 5 × 10^5^/mL in triplicate. T24 cells were harvested and washed with cooled PBS. Nuclear and cytoplasmic extractions were isolated using the Active Motif^®^ Nuclear Extraction Kit, following the manufacturer’s instructions. For total protein, cells were washed twice with ice-cold PBS, incubated in 20 mM Tris-HCl buffer (pH 8.0), 2 mM EDTA, 150 M NaCl, and 10% glycerol; 7 × protease inhibitor solution for 30 min at 4 °C. A sterile cell scraper was used to scrape the cells off the plate, gently transferred into pre-cooled Eppendorf tubes, centrifuged at 13,000× *g*, for 15 min at 4 °C. Protein-containing supernatant was collected for each sample and stored at −80 °C. The protein concentration was determined by Brilliant Blue G dye-binding assay of Bradford using bovine serum albumin as a standard.

The protein extractions (10–40 μg) were run in 10% SDS-polyacrylamide gel electrophoresis (SDS-PAGE). The resolved protein bands were transferred onto PVDF membranes (Bio-Rad, Hertfordshire, UK) using a semi-dry transfer system. The membranes were blocked with 5% fat-free dry milk in PBS (pH 7.4) containing 0.1% Tween-20 for 30 min at room temperature, followed by incubation with primary antibodies (working dilution 1:1000) in PBS overnight at 4 °C. The targeted protein was visualized with an enhanced chemiluminescent system (GE Healthcare, Little Chalfont, UK) or Odyssey system according to the manufacturer’s instructions.

### 4.7. HPLC Analysis of Intracellular GSH

T24 cells were seeded in six-well plates at 1 × 10^5^ cells/well in a final volume of 3 mL and then were treated with SFN (5–20 μM) or vehicle DMSO (0.1% as control). After treatment, cells were counted and collected. Then, cells were resuspended in 75 μL diethylenetriaminepentaacetic acid (DPTA) and 300 μL of 50 mM methanesulfonic acid (MSA), and transferred into 1.5 mL Eppendorf tubes and stored in −80 °C. The samples (GSH-containing supernatants) were subjected to three freeze-thaw cycles alternating between −80 °C and 37 °C heat block for 6 min each time and vortexed, then collected the supernatant after centrifuged. Quantification was performed using Bradford assay. To compare the results with an accurate standard curve, four standards samples (20, 10, 5, 1.25 µg/mL) were prepared from a 1 mg/mL GSH stock solution. A premix buffer (25 μL), prepared by mixing 10 µL of 0.5 M HEPEs, 1 µL 0.5 M EDTA, 1.5 µL of 1 M NaOH, 2 µL of 0.1 M mBBr, and 10.5 µL of Acetonitrile (100%), was added to 75 μL of each sample, vortexed immediately and incubated for 15 min in the dark at room temperature. MSA was added to acidify the samples. GSH-mBBr adduct was measured by HPLC, chromatographic separating was achieved using a Synergi Hydro-RP vs. Luna^®^ C18 4.6 × 150 mm, 5 μm, 100 A column (Phenomenex) equilibrated at 37 °C with solvent A: 90% Milli Q water in methanol containing 0.25% (*v/v*) acetic acid, adjusted to pH 4 with NaOH. Samples were eluted with a gradient of Solvent B (90% Methanol) at a 1.0 mL/min flow rate as follows: 0–10 min, 0% Solvent B; 10–11 min, 50% Solvent B; and 11–15 min, 100% Solvent B, 16–20 min, 0% Solvent B; followed by equilibration and re-injection. Detection was carried out with a Jasco fluorescence detector with excitation at 385 nm and emission at 460 nm, and a gain of 1 × GSmB eluted at 7.1 min, and was quantified against a standard curve. The level of GSH was expressed as nmol/mg of cellular soluble protein.

### 4.8. Knockdown Gene by siRNA

The siRNAs of Nrf2 and γ-GCS sequences, were obtained from Applied Biosystems. T24 cells were seeded in 6 well plates at 2 × 10^5^ cells/well in a final volume of 3 mL of growth medium, then transfected with siRNA (10 nM) for Nrf2 or AllStars (negative control, that has no homology to any known mammalian gene) for 24 h following the manufacturer’s instructions.

### 4.9. Statistical Analysis

Data were represented as the mean ± SD. One-way ANOVA with Tukey’s post hoc analysis was used to assess multiple groups when all or many groups pairwise comparisons were of interest. A *p* value < 0.05 as considered statically significant.

## Data Availability

The data presented in this study are available upon request to the author C.H.

## Sample Availability

Samples of the compounds are not available from the authors.
